# Investigating autism knowledge, self-efficacy, and confidence following maternal and child health nurse training for the early identification of autism

**DOI:** 10.3389/fneur.2023.1201292

**Published:** 2024-01-09

**Authors:** Katherine Gore, Melissa Gilbert, Marguerite Hawke, Josephine Barbaro

**Affiliations:** Olga Tennison Autism Research Centre, School of Psychology and Public Health, La Trobe University, Bundoora, VIC, Australia

**Keywords:** autism, early identification, early detection, infants, maternal and child health nurses, toddlers, workplace training

## Abstract

**Introduction:**

Early identification of children with a high likelihood of autism can lead to referral for diagnostic services and access to early supports, resulting in improved outcomes for children and families. Maternal and Child Health Nurses (MCHNs) in Victoria, Australia, are well-placed to monitor infants and toddlers for signs of autism, given children and caregivers attend free, regular, well-baby consultations from birth through to school age. This study aimed to identify the impact of personal and workplace factors on MCHNs’ competencies of autism knowledge, self-efficacy in identifying autistic infants and toddlers, and confidence in speaking to parents/caregivers about autism. Additionally, the study sought to identify which personal and workplace factors might predict increased competency in these areas.

**Methods:**

After identifying training needs and current competency levels via a training needs analysis (TNA), 1,428 MCHNs received training on the early signs of autism and in the use of the Social Attention and Communication Surveillance-Revised (SACS-R) tool for early autism identification; the training program was known as Monitoring of Social Attention, Interaction, and Communication (MoSAIC).

**Results:**

Previous MCHN autism training and knowledge of autism community resources significantly contributed to increased MCHN self-efficacy in identifying autistic infants and toddlers, while knowledge of community resources was the best predictor of confidence in speaking with parents/caregivers about autism. Perceived self-efficacy and confidence in speaking with parents/caregivers about autism significantly increased following the MoSAIC autism training.

**Discussion:**

Targeted autism training for primary health practitioners is an important first step for early autism identification and initiating conversations with parents/caregivers.

## 1 Introduction

In Australia, professionals typically involved in the early identification of autistic children include general practitioners (GPs; or primary care physicians), pediatricians, speech pathologists, psychologists, maternal and child health nurses (MCHNs), and early childhood educators (1). Early identification of children with a high likelihood for autism leads to early diagnosis and access to early supports and services, which may result in improvements in cognitive ability and better overall outcomes for children and families (2–6). However, a lack of knowledge of the early signs of autism and low self-confidence in identifying autism, limits professional capability (7, 8). Fortunately, training has been shown to improve autism knowledge, self-efficacy, and screening rates (9–11).

Recent research has focused on the developmental surveillance capabilities of professionals who have regular contact with infants and toddlers, given the critical role they play in the pathway to early supports and services (12–14). In Victoria, Australia, the Maternal and Child Health (MCH) service is free and universally available, and parents/caregivers can attend regular MCH ‘key ages and stages’ well-baby consultations with their child from birth through to school age. Therefore, MCHNs are in prime position to be involved in the early identification of autism in infants and toddlers and to support their caregivers (15). Consultations cover developmentally appropriate milestones and guidance, including whether the child is meeting their social-communication milestones (15). Ninety-six percent of parents/caregivers access the MCH service when their child is 2 weeks old and attendance remains at high levels, with 70% attendance at 24 months of age (16). Similar child health services with high utilization are found both nationally (17) and internationally (18–20).

Previous studies have demonstrated the benefit of focusing on MCHN screening/developmental surveillance for the early identification of autistic children (20–22). The effectiveness of MCHN developmental surveillance for autism, when enhanced by training in typical and atypical social communicative development, was established in The Social Attention and Communication (SACS) Study (3, 23). A total of 241 MCHNs, at 170 MCH Centers, in 17 local government areas in metropolitan Melbourne, Victoria, were trained in key social communication milestones and early autism developmental surveillance using the SACS tool (23). Children found by their MCHN to have a high likelihood for autism were referred to the study team for further investigation. The SACS tool was found to be highly accurate in community-based early autism screening, with a positive predictive value of 83% (23). A subsequent study indicated that the revised SACS tool (SACS-R) had a positive predictive value of 83% for children aged 11–30 months old, and that when an additional SACS preschool tool (SACS-PR) was added at their 42-month MCH check (range: 31–60 months), sensitivity increased to 96% (24).

### 1.1 Evaluating changes in competency levels after autism specific training

In the initial SACS training study by Barbaro and colleagues (23) it was established that 99% of MCHNs strongly agreed that they felt able to monitor the early signs of autism immediately after training. They also reported high levels of confidence (82–97%) in using SACS at two timeframes – 6-9 months after the study commenced, and in a final survey distributed at the conclusion of the research study. A 2022 study by Shrestha and colleagues (25), reviewed the training of 60 Nepalese Female Community Health Volunteers (FCHVs) in the early signs of autism and use of the SACS-R tool, and found significant improvements in FCHVs’ autism knowledge and confidence in monitoring autism in young children, with improvements still evident one-year post-training. Waddington and colleagues also found that well-child/Tamariki Ora nurses in New Zealand were more confident discussing autism observations with parents after SACS-R training (10).

Wider research has evaluated training on autism knowledge and other autism screening tools. Studies involving other healthcare professionals have demonstrated improvements in autism knowledge (11, 12, 14, 26, 27), increased self-efficacy in screening for autism in children (11, 12, 26, 28, 29), increased rates of autism screening (29–31), and greater confidence in discussing autism with families (26, 27, 30, 31).

Though it is clear that MCH (and equivalent) nurses can play an important role in the early identification of autism, there has been little in-depth analysis of factors that influence their autism knowledge and ability to support young children identified as having a high likelihood of autism, and their families. Therefore, an opportunity to better understand predictors of these competencies and whether autism specific training improves performance in these areas, was identified.

### 1.2 Predictors of autism knowledge

No known previous research has examined predictors of autism knowledge in MCH or equivalent nurses, though this has been explored in other healthcare professional populations with mixed findings. Age has been studied as a predictor variable in several healthcare populations, with mixed results. Studies of Australian GPs found an inverse relationship between age and autism knowledge (1), whereas a study of Italian pediatric nurses found a direct relationship, which might be explained by their expertise in the development of infants and toddlers (32). Other studies with samples of UK GPs, UK psychiatrists, Singapore GPs, and US school psychologists observed no relationship between age and autism knowledge (33–37). Examination of the effect of years of practice has similarly yielded mixed results (1, 32, 33, 36).

Other potential predictors previously studied include knowledge of community resources for autism (1) and having a personal connection with an autistic person, such as a friend or family member (33, 36), both of which were positively correlated with autism knowledge levels. The role of knowledge of community resources for autism as a predictor may be explained by community resources playing an educative role, as well as a supportive and/or therapeutic role, which can help build health professionals’ skills in identifying autistic children.

The findings regarding prior autism training and autism knowledge were mixed. Number of hours of autism training in the past five years was found to be correlated with increased autism knowledge in a sample of US school psychologists (35). However, other studies involving US school psychologists, UK psychiatrists, and UK GP populations found no relationship between prior autism training (during their degree and ongoing education) and autism knowledge (33, 34, 36). A final predictor of autism knowledge considered in the literature was the potential impact of metropolitan versus rural location, and no significant correlation was found (34).

### 1.3 Predictors of self-efficacy in identifying autism

No known previous research has examined predictors of self-efficacy in identifying autistic children in MCH or equivalent nurses, though this has been explored in other healthcare professional populations with mixed findings (30, 31). Self-efficacy, in this context, is self-belief in one’s own ability to identify the signs of autism in children.

When considering age and years of practice, a study of 367 US neuropsychologists, at varying points in their careers, found no effect of either age or years of practice on confidence in identifying autism (38). However, in a sample of UK GPs, years of experience were positively associated with higher self-efficacy levels (39).

Prior autism training (during degree and ongoing education) was found to positively predict self-efficacy in identifying and supporting autistic children (33, 36, 39, 40). Self-efficacy in identifying the signs of autism in children is required by healthcare professionals to successfully administer autism screening and referrals; therefore, increased screening may be indicative of increased self-efficacy. Rural family practices in the US were found to have lower autism screening rates than those in urban locations (5), which could suggest lower levels of self-efficacy in regional areas.

### 1.4 Confidence in speaking about autism to parents/caregivers

There is limited literature on health professionals’ confidence in discussing children’s autism ‘likelihood’ with parents/caregivers. Predictors of healthcare professionals’ confidence appear to have been considered when evaluating autism training effectiveness (30, 31). However, a 2021 study of 357 practicing nurses in Palestine found that only 9% of nurses felt confident counseling parents about autism (41). A moderate positive correlation was found between autism knowledge and confidence in discussing autism with parents, as well as a significant relationship between years of practice and continuing education on autism and increased knowledge. Taken together, these findings suggest that when nurses are more experienced and better educated on the early signs of autism, they are more confident in discussing early autism signs with parents.

### 1.5 The current study

In September 2018, the Victorian State Government committed funding to train the Victorian MCH workforce in the early identification of autism (42). The Government also selected the SACS-R tool, the most accurate autism screening tool at the time, and the training included the use of SACS-R to enable MCH workers to monitor all children for the early signs of autism (42). The Victorian MCH workforce comprises MCH nurses, casually-employed relief MCH nurses, students, telephone counselors, managers, site co-ordinators, team leaders, Aboriginal Community organization nurses, early parent center nurses, home visiting (‘right@home’ and ‘Enhanced Home Visit’) nurses, and lactation consultants.

**Figure 1:**
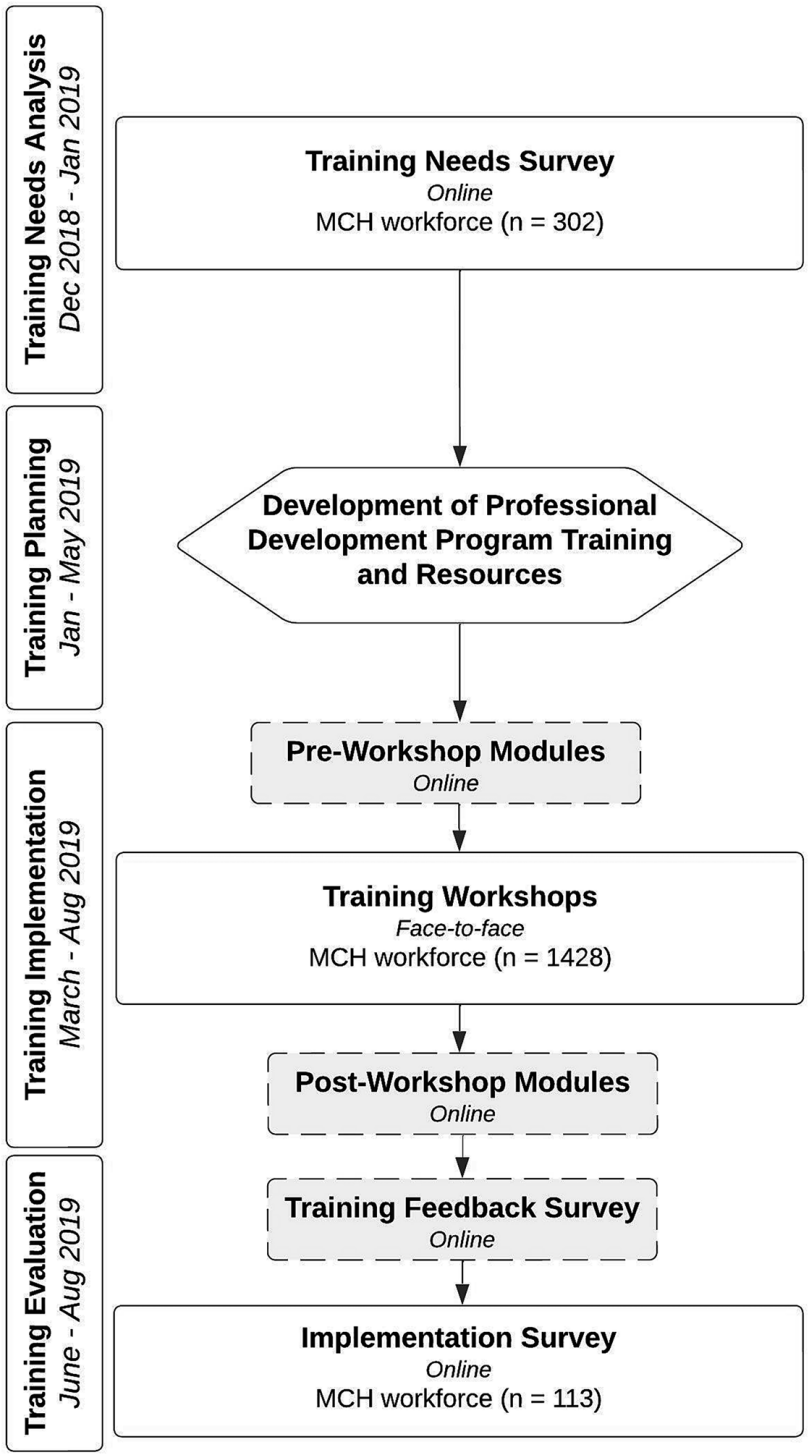
MoSAIC training flowchart. Figure elements with dotted lines were part of the MoSAIC training process but were not the focus of analysis in this current paper. For further detail on these elements of the training process see Gilbert et al. (43).

The Olga Tennison Autism Research Centre (OTARC) was selected to design and deliver this training program. A Training Needs Analysis (TNA) was conducted by OTARC on behalf of the Victorian State Government, to identify key information relating to the experiences of MCH nurses and the parents/caregivers they served, as well as learnings from a group of autistic adults. The TNA comprised of (1) four focus groups, (2) a referral pathways workshop, and (3) a training needs survey (TNS), which sought information about the participant’s demographic, workplace, autism knowledge, experience of autism screening/developmental surveillance, and perceived training needs. The results of the TNA indicated that autism knowledge, the ability to identify infants and toddlers at high likelihood for autism, and confidence in communicating with parents/caregivers about autism, were key competencies to be covered in the training. These findings were used to develop the professional development program delivered to the MCH workforce, called Monitoring of Social Attention, Interaction, and Communication (MoSAIC) (43), which included training in the use of the SACS-R tool. Training evaluation comprised (1) a training feedback survey, (2) an implementation survey, and (3) a parent evaluation survey, of which only the implementation survey is analyzed in this current article. Figure 1 details the stages of training development and evaluation.

In the current paper, we describe the TNS – a survey created to measure competency and identify gaps in MCHN autism knowledge and identification of autism in children - prior to the MoSAIC training, as well as the implementation survey, which was used to assess changes in key competencies after training.

### 1.6 Study aims and hypotheses

The aims of the current study were to identify the impact of various personal and employment-related factors on Victorian MCHNs’ autism knowledge. The influence of age, years of experience, location, autism training, knowledge of community resources, and a personal connection with an autistic person were examined. A further aim was to identify the impact of these variables on MCHN self-efficacy in identifying infants and toddlers at high likelihood for autism, and confidence in speaking to parents/caregivers about autism.

The study also aimed to determine which of these variables would predict autism knowledge, self-efficacy in identifying infants and toddlers at high likelihood for autism, and MCHN confidence in speaking with parents/caregivers about autism. A final aim was to compare MCHNs who responded to both the TNS and the implementation survey, to determine whether there was significant change in their autism knowledge, self-efficacy in identifying infants and toddlers at high likelihood for autism, and their confidence in speaking to parents/caregivers about autism.

It was hypothesized that there would be a significant relationship between MCHN age, years of experience, location, amount of previous autism training, knowledge of autism community resources, and having a personal connection with an autistic person; and MCHN autism knowledge, self-efficacy in identifying early autism signs, and confidence in speaking to parents/caregivers about autism. The second hypothesis was that the amount of previous training would predict greater MCHN autism knowledge, self-efficacy, and confidence. The final hypothesis was that MCHNs would have significant increases in autism knowledge, self-efficacy, and confidence after completing the MoSAIC training.

## 2 Materials and methods

### 2.1 Participants

#### 2.1.1 MCHN workforce training needs survey (TNS)

The survey link was sent to the email addresses of members of the MCH workforce supplied by the Victorian Department of Health and Human Services on 10 December 2018, which included MCH nurses, students, telephone counselors, managers, coordinators, team leaders, and other related roles (*n* = 1,428). The TNS had 350 respondents (a 25% response rate), with a final sample of 302 participants after exclusion criteria were applied; these included two student nurses and 16 participants who did not conduct well-baby checks in their roles; 28 participants who only completed approximately one-third of the survey; and two participants who completed the survey twice, with their second response deleted.

#### 2.1.2 MCHN workforce autism training

All participants were part of a Victorian Government funded project to provide mandatory training to the Victorian MCHN workforce to increase the early identification of autism (*n* = 1,510). The MCHN workforce training, hereafter referred to as the MoSAIC training, covered early social-communication differences in autistic children, how to use the SACS-R tool, how to initiate and provide referrals to healthcare professionals for further assessment (e.g., GPs, pediatricians, and psychologists) and referrals to early supports and services. The training also included a module on how to discuss autism with parents/caregivers. All respondents were female, over 18 years of age, and from a range of metropolitan, rural, and regional locations across Victoria. Given the very small number of male MCHNs in Victoria (44), it was not unexpected that all respondents were female.

#### 2.1.3 Assessing competency changes – pre- and post-training

Only those participants who had completed the TNS were eligible for means comparison between pre-and post-training (*n* = 38). Therefore, while 344 participants completed the implementation survey after the MoSAIC training, there was only matched pre-and post-training data available for 38 MCHNs.

### 2.2 Materials

Surveys for each component of the study were hosted on the Qualtrics survey platform (45).

#### 2.2.1 Training needs survey

The TNS included 142 questions across six sections: ‘Demographics’, ‘About your workplace and role’, ‘About your autism knowledge and practice’, ‘About developmental surveillance in autism’, ‘Your current training needs’, and ‘Tell us more’. Two pre-existing surveys were modified for inclusion in this survey (i) The Determinants of Implementation Behavior Questionnaire (DIBQ) (46), and (ii) an autism knowledge survey, adapted from Shrestha et al. and Waddington et al. (10, 25).

The DIBQ (45), based on the Theoretical Domains Framework (47), captures factors that can influence change behavior when new healthcare practices are implemented after training. Two DIBQ domains (Domain 4 and Domain 15) were adapted for use in the TNS. Two items were adapted from “Domain 4: Beliefs about capabilities” – the first asked participants to respond to statements regarding their abilities in identification and monitoring signs of autism on a five-point Likert scale (from “Strongly Agree” to “Strongly Disagree”), and the second asked participants to rate their experience of monitoring atypical social communication skills (also on a five-point Likert scale from “Very Difficult” to “Very Easy”). Four items were adapted from “Domain 15: Positive emotions” which asked participants to rate how they felt about monitoring early social communication and autism on seven-point semantic differential scales for the following adjectives: “optimistic” (versus “pessimistic”), “comfortable” (versus “uncomfortable”), “calm” (versus “anxious/apprehensive”), and “relaxed” (versus “stressed”).

The autism knowledge survey comprised of 39 items relating to knowledge of early childhood social communication and the early signs of autism (48–50), and was found to have acceptable internal reliability (α = 0.76).

#### 2.2.2 Implementation survey

The detailed implementation survey included 127 questions across six sections: ‘About your autism knowledge and practice’, ‘Monitoring atypical social communication skills’, ‘Monitoring of social attention, interaction, and communication (MoSAIC)’, ‘Your experience using MoSAIC in practice’, ‘Supporting you to use MoSAIC’, and ‘Tell us more’. The survey addressed several areas, including MCHN experiences using MoSAIC in their practice and their current knowledge about autism and social communication behavior. Several items included in the TNS were included again in this survey, to assess change from pre-to post-training, such as those relating to autism knowledge, skills in the early identification of children at high likelihood of autism, and confidence in speaking with parents/caregivers about autism. The autism knowledge survey sections were again found to have acceptable internal reliability for the post-training survey (α = 0.78).

### 2.3 Procedure

#### 2.3.1 Training needs survey (TNS)

An email with a link to the TNS, the participant information statement, and consent forms, was sent to addresses belonging to the MCHN workforce (*n* = 1,428), supplied to the research team by the Victorian Department of Health and Human Services. The survey opened on 11 December 2018 and closed on 30 January 2019, with reminders emailed every 2 weeks over the survey duration.

#### 2.3.2 Training needs survey/implementation survey matched sample

A detailed implementation survey was sent to members of the MCHN workforce (*n* = 1,510) 4 to 6 weeks after they attended the MoSAIC training, using the initial email address provided on registration. The implementation surveys were completed between 1 July and 31 August 2019 by 133 MCHNs. To create a within-groups cohort for analysis, it was necessary to match participants from the TNS with the 113 MCHNs who completed the implementation survey using name and email address. Based on a power analysis using G*Power 3.1.9.4 (51) for a within subjects group design, with a medium effect size (0.5) and an alpha level of 0.05 for statistical significance, a minimum of 34 participants were required for this study after the matching process.

### 2.4 Statistical analyses

In the TNS sample and TNS/implementation survey matched sample, the socio-economic status (SES) of each MCHN’s work location was calculated by mapping their primary work location Local Government Area against the Australian Socio-Economic Indexes for Areas (SEIFA) (51). Descriptive analyses were completed, with a chi-square goodness-of-fit test performed on the demographics. This found the “low SES” category, which had fewer than five participants in the matched sample, was significantly different to the TNS sample. However, it was not collapsed into other categories when assessing the TNS/implementation survey matched sample as SES level could be meaningful, with a Fisher’s exact test conducted instead.

#### 2.4.1 Training needs survey (TNS)

Standardization of values to Z-scores revealed four extreme univariate outliers for the autism-knowledge variable. All outliers had negative Z-scores and were winsorized to one unit more than the next most extreme score (52). Reflect and inverse transformation (52) of the autism-knowledge variable reduced skewness and kurtosis. Reflect and logarithmic transformation (52) of age group and years of practice improved normality. The personal connection with an autistic person variable did not respond to transformation. The three dependent variables passed all multiple regression assumption testing.

TNS analysis began with Kendall’s tau-b correlation analyses for the ordinal independent variables of age, years of practice, and autism training; point-biserial correlation for the dichotomous independent variables of work location and personal connection to autism; and Pearson’s correlation for the continuous independent variable of knowledge of autism community resources. Multiple regression followed using the independent variables that had significant correlations to determine the strongest predictors of competency in autism identification.

#### 2.4.2 Assessing competency changes: TNS/implementation survey matched sample

Standardization of difference values to Z-scores revealed one extreme univariate in the autism-knowledge variable in the TNS. A decision was made to winsorize this score to one unit less than the next most extreme score (52) which improved kurtosis and normality. To assess competency changes, a paired samples t-test determined if there were significant changes in mean scores for the three dependent variables.

## 3 Results

### 3.1 Sample description

Table 1 presents the demographic characteristics in the TNS sample and the TNS/implementation survey matched sample. Most of the TNS sample (78.47%) and the matched sample (86.84%) were aged over 46 years. Both cohorts were well-educated, with 90.73% and 89.47%, respectively, having a postgraduate degree, and both samples being highly experienced MCHNs, with 49.67% and 42.11%, respectively, having worked more than 10 years as an MCHN.

**Table 1:** Demographic characteristics of training needs survey (TNS) sample and TNS/implementation survey matched sample.

	TNS sample		TNS/Implementation Survey matched sample	
	(*N* = 302)		(*N* = 38)	
Characteristic	n	%	n	%
Gender				
Female	302	100	38	100
Male	0	0	0	0
Indeterminate/intersex/unspecified	0	0	0	0
Age range (years)				
26–35	18	5.96	2	5.26
36–45	46	15.23	3	7.89
46–55	105	34.76	16	42.11
56 or over	132	43.71	17	44.73
Prefer not to say	1	0.33	0	0
Highest level of completed education				
Certificate, hospital qualification, or diploma	15	4.97	3	7.89
Undergraduate Degree/s	13	4.3	1	2.63
Postgraduate Diploma/s	205	67.88	25	65.79
Master’s Degree/s	68	22.52	9	23.68
Doctoral Degree/s	1	0.33	0	0
Years of practice as a nurse				
Less than 3 years	34	11.26	2	5.26
3–5 years	44	14.57	6	15.79
6–10 years	74	24.5	14	36.84
11–15 years	43	14.24	4	10.53
More than 15 years	107	35.43	12	31.58

Table 2 presents the MCHN workforce characteristics of the TNS sample and the TNS/implementation survey matched sample. More than half of the participants worked less than 30 hours a week, over 60% were based in metropolitan locations, with few working in the two most socio-economically disadvantaged locations (3.82 and 2.61%, respectively). The results of a Fischer’s exact test were not significant, indicating that the proportions of locations based on SES were not significantly different between the two samples.

**Table 2:** Workplace characteristics of training needs survey (TNS) sample and TNS/implementation survey matched sample.

	TNS sample		TNS/Implementation survey matched sample	
	(*N* = 302)		(*N* = 38)	
Characteristic	n	%	n	%
Hours worked per week in current role(s)				
Less than 15 h	32	10.6	7	18.42
16–25 h	98	32.45	8	21.05
26–30 h	55	18.21	7	18.42
More than 30 h	117	38.74	16	42.11
Current role(s)				
MCH center nurse	221	73.18	26	68.42
MCH nurse reliever	30	9.93	4	10.53
MCH nurse team leader/manager/site coordinator	17	5.63	2	5.26
Enhanced home visiting MCH nurse	39	12.91	5	13.16
Aboriginal Controlled Community organization MCH nurse	4	1.32	1	2.63
MCH Health Line Telephone Counselor	11	3.64	1	2.63
Early parenting center MCH nurse	7	2.32	2	5.26
Other – MCH right@home nurse, MCH and UMCH, lactation consultant	6	1.99	2	5.26
Primary work location^#^				
Metropolitan	206	71.53	23	60.52
Rural city or regional	82	28.47	12	31.58
Other/Not applicable	0	0	3	7.89
Socio-economic status of work location^#^				
1 (bottom 20% most disadvantaged nationally)	11	3.82	1	2.63
2–4	164	56.95	23	60.53
5 (top 20% most advantaged)	113	39.24	11	28.95
Not applicable	0	0	3	7.89

Respondents could select more than one answer when selecting their current role. Where respondents had more than one role, they were asked to provide the main Local Government Area that they worked in. MCH, Maternal & Child Health. UMCH, Universal Maternal & Child Health. ^#^In the Training Needs Survey sample, 14 nurses did not provide their primary work location. The percentage analysis for primary work location for the Training Needs Survey sample is based on *N* = 288.

### 3.2 Relationships between MCHN age, experience, location, previous autism training, community resource knowledge; and personal connection with an autistic person and MCHN autism knowledge, self-efficacy, and confidence

#### 3.2.1 MCHN age

As presented in Table 3, there was a small, negative association between age and self-efficacy in identifying children at high likelihood for autism, *τ_b_* = −0.12, *p* = 0.01 and a small, negative association between age and confidence in discussing autism with parents/caregivers, *τ_b_* = −0.11, *p* = 0.02. The association between age and autism knowledge was not significant.

**Table 3:** Summary of correlations for the relationships between independent variables and dependent variables in the training needs survey sample.

	Autism knowledge (*N* = 302)			Self-efficacy in identifying children at high likelihood of autism (*N* = 294)			Confidence in discussing autism with parents/caregivers (*N* = 294)		
Variable	*τ_b_*	*p*	*ES*	*τ_b_*	*p*	*ES*	*τ_b_*	*p*	*ES*
Age	0.003	0.95		−0.12	0.01*	0.01	−0.11	0.02*	0.01
Years of practice as a MCHN	−0.003	0.95		−0.20	<0.001*	0.04	−0.20	<0.001*	0.04
Amount of autism training	0.11	0.01*	0.01	0.43	<0.001*	0.19	0.28	<0.001*	0.08
	*r_pb_*	*p*	*ES*	*r_pb_*	*p*		*r_pb_*	*p*	*ES*
Location^	−0.03	0.66		−0.07	0.24		−0.13	0.03*	0.02
Personal connection with an autistic person^#^	0.12	0.03*	0.01	0.06	0.34		0.09	0.11	
	*r*	*p*		*r*	*p*	*ES*	*r*	*p*	*ES*
Knowledge of community resources for autism	0.10	0.10		0.34	<0.001*	0.12	0.39	<0.001*	0.14

Autism knowledge is based on 294 participants for the knowledge of autism community resources independent variable and 302 participants for all other independent variables. Eight participants left the survey after the autism knowledge questions and did not complete questions relating to the knowledge of autism community resources for autism and did not complete questions relating to the other dependent variables of self-efficacy and confidence.^For location, 1, metropolitan; 2, regional/rural area.^#^For personal connection with an autistic person: 0, no connection; 1, personal connection.*significant. τ_b =_ Kendall’s tau-b. *r* = point biserial correlation.

#### 3.2.2 MCHN years of practice

There was a small, negative association between years of practice and self-efficacy in identifying children at high likelihood of autism, *τ_b_* = −0.20, *p* < 0.001 and a small, negative association between years of practice and confidence discussing autism with parents/caregivers, *τ_b_* = −0.20, *p* < 0.001. The association between years of practice and autism knowledge was not significant.

#### 3.2.3 Amount of previous autism training

Previous amount of autism training had a small, positive correlation with autism knowledge, *τ_b_* = 0.11, *p* = 0.01, a medium, positive correlation with self-efficacy, *τ_b_* = 0.43, *p* < 0.001, and a small, positive correlation with confidence discussing autism with parents/caregivers, *τ_b_* = 0.28, *p* < 0.001.

#### 3.2.4 Work location

There was a small, negative correlation between work location and confidence discussing autism with parents/caregivers, $r_{pb}$ = −0.13, *p* = 0.03, indicating confidence levels are lower for MCHNs working in rural and regional areas. There was no significant correlation with autism knowledge or with self-efficacy.

#### 3.2.5 Personal connection with an autistic person

There was a small, positive correlation between personal connection with an autistic person and autism knowledge, $r_{pb}$ = 0.12, *p* = 0.03. There was no significant correlation with self-efficacy or with confidence discussing autism with parents/caregivers.

#### 3.2.6 Knowledge of autism community resources

There was a medium, positive correlation between knowledge of autism community resources and self-efficacy in identifying children at high likelihood for autism, *r*(292) = 0.34, *p* < 0.001 and a medium, positive correlation with confidence in discussing autism with parents/caregivers, *r*(292) = 0.39, *p* < 0.001. There was no significant correlation with autism knowledge.

### 3.3 Predictors of autism knowledge, self-efficacy, and confidence

#### 3.3.1 Autism knowledge

In combination, previous autism training and personal connection with an autistic person accounted for statistically significant variability (4%) in MCHN autism knowledge, *R*^2^ = 0.04, adjusted *R*^2^ = 0.03, F(2,299) = 5.91, *p* = 0.003, with a small effect (*d* = 0.04; see Table 4). The most significant contributor was previous autism training, with 2% of unique variance in autism knowledge attributed to this predictor.

**Table 4:** Summary of multiple regression analysis predicting MCHN competency in the training needs survey sample.

	Autism knowledge levels			Self-efficacy identifying children at high likelihood of autism			Confidence in speaking with parents/caregivers about autism		
Variable	*B* [95% CI]	ß	𝑠𝑟2	*B* [95% CI]	ß	𝑠𝑟2	*B* [95% CI]	ß	𝑠𝑟2
Age group				0.007 [−0.50, 0.490]	0.002	<0.001	0.01 [−0.66, 0.68]	0.002	<0.001
Years of practice				−0.22 [−0.57, 0.13]	−0.08	0.004	−0.49*[−0.97, −0.02]	−0.14	0.01
Amount of autism training (self-report)	0.03* [0.007, 0.05]	0.15	0.02	0.20** [0.15, 0.25]	0.41	0.15	0.12**[0.06, 0.19]	0.20	0.04
Personal connection with an autistic person	0.08* [0.001, 0.16]	0.11	0.01						
Work location							−0.24* [−0.46, −0.03]	−0.12	0.01
Knowledge of community resources for autism				0.16** [0.09, 0.24]	0.21	0.04	0.29**[0.19, 0.39]	0.30	0.09

*p < 0.05; **p < 0.001; CI, Confidence Interval.

### 3.4 Self-efficacy

In combination, age group, years of practice, training, and knowledge of autism community services accounted for statistically significant variability (28%) in MCHN self-efficacy in identifying children at high likelihood for autism, *R*^2^ = 0.28, adjusted *R*^2^ = 0.27, F(4,289) = 28.50, *p* < 0.001, with a small effect (*d* = 0.40; see Table 4). The most significant contributor was previous autism training, with 15% of unique variance in nurse self-efficacy attributed to this predictor.

### 3.5 Confidence levels

In combination, age group, years of practice, training, location, and knowledge of autism community services accounted for 23% of variability in MCHN confidence in talking to parents/caregivers about autism, which was significant; *R*^2^ = 0.23, adjusted *R^2^ =* 0.22, F(5, 274) = 16.38, *p* < 0.001, with a small effect (*d* = 0.33; see Table 4). The most significant contributor was knowledge of community resources for autism, with 9% of unique variance in nurse confidence attributed to this predictor. All predictors except for ‘age group’ made a significant contribution to confidence levels.

### 3.6 Competency changes pre/post-training on MoSAIC

The difference in mean scores for overall autism knowledge pre-training (*M* = 35.90, *SD* = 3.37) and post-training (*M* = 36.84, *SD* = 2.78) was not statistically significant *t*(37) = 1.48, *p* = 0.15. However, the difference in mean scores for perceived self-efficacy levels in identifying infants and toddlers at high likelihood for autism pre-training (*M* = 3.97, *SD* = 0.60) and post-training (*M* = 4.39, *SD* = 0.72) was statistically significant *t*(37) = 3.42, *p* = 0.002, with medium effect, *d* = 0.64. Furthermore, the difference in mean scores for confidence levels in speaking to parents/caregivers about autism pre-training (*M* = 3.34, *SD* = 0.97) and post-training (*M* = 3.95, SD = 0.77) was statistically significant, *t*(37) = 4.21, *p* < 0.001, with medium effect, *d* = 0.70.

## 4 Discussion

### 4.1 Autism knowledge

High amounts of previous autism training and having a personal connection with an autistic person were correlated with higher autism knowledge levels and this finding is consistent with previous research (33, 35, 36). This could be explained by the notion that individuals who have a personal connection may feel more motivated to undertake training about autism (53, 54). The relationship between training and knowledge is not unexpected, given previous autism training with MCHNs focused on increasing their knowledge and competency in autism screening.

Higher amounts of previous autism training made the greatest significant unique contribution to variance in autism knowledge, followed by personal connection to an autistic person. The overall model had a small effect, suggesting there may be other variables which better explain variance in autism knowledge in the MCHN workforce, which should be explored in future research.

The lack of significant increase in overall autism knowledge levels after autism specific training differs from other research findings involving Western healthcare practitioners such as physicians, pediatricians, and physical therapists (12, 14, 30, 31), as well as well-child/Tamariki Ora nurses in New Zealand and Female Community Health Volunteers in Nepal, who saw improvements in knowledge after training (10, 25), using the same training program detailed in this paper. This may be explained by the already high level of autism knowledge within the MCHN workforce cohort before MoSAIC training began, or it could potentially be explained by self-selection bias whereby MCHNs with high levels of autism knowledge and/or MCHNs who did not feel the training changed their knowledge levels, were more motivated to complete the surveys. Given the small effect size for the correlation and regression results and the high negative skew in the autism knowledge variable, which indicated a high level of knowledge already existed in the cohort, these findings should therefore be interpreted with caution.

Future research could refine this questionnaire by focusing more on the early presentation of autism rather than overall autism knowledge, validating the autism knowledge questionnaire in other populations, and conducting principal component analysis to better understand the questionnaire’s structure.

### 4.2 Self-efficacy

Results indicated a significant negative relationship between age and years of practice with self-efficacy, as well as a significant positive relationship between autism training and knowledge of community resources for autism, and self-efficacy in identifying children at high likelihood for autism. A possible explanation for the finding relating to age and years of practice is that some older and more experienced members of the workforce may feel less confident in learning about, and utilizing, new tools and methods, particularly if they are computer-based, as per the SACS-R tool. For example, past research has found that older and more experienced nurses may find it more challenging to learn about new computer-based systems and to cope with changes that may add complexity to their roles (55–57).

Prior autism training and knowledge of community resources for autism both made significant contributions in explaining MCHN self-efficacy levels in identifying infants and toddlers at high likelihood for autism. There was also an increase in mean levels of self-efficacy after the MoSAIC training, which supports previous research on increased self-efficacy following autism specific training (12, 25, 30). This could be explained by autism training focusing on both screening tools and general knowledge about autism.

### 4.3 Confidence in talking to parents/caregivers about autism

Less experience in current workplace role and younger age were significantly correlated with higher confidence in talking to parents/caregivers about autism. MCHNs in metropolitan locations also had higher confidence levels than those in rural and regional areas. There was a positive relationship between knowledge of community resources for autism and confidence. Knowledge of community resources for autism was the best predictor of confidence, with location, years of practice, and training also making significant unique contributions. There was also an increase in mean levels of confidence in speaking with parents/caregivers about autism, post-training, which was consistent with previous research evaluating autism surveillance training in other healthcare worker populations (10, 25, 30, 58).

Previous research has proposed that increased content on autism in university courses, in more recent years, may explain why younger age is correlated with increased autism knowledge (1), an explanation that could also extend to confidence. Another possible explanation for this finding is that younger nurses may feel more comfortable with talking about autism given its increased profile in contemporary culture through the media, film, television, and social media (59–61). The finding that knowledge of community resources for autism is a significant predictor of confidence could be explained by nurses feeling more confident to speak with parents/caregivers when they feel more prepared to answer expected questions, such as how to get a formal diagnosis, or access supports and services in the local community.

The finding that working in a metropolitan location has a positive influence on MCHNs’ confidence in speaking to parents/caregivers about autism is important to consider, given Australia’s geography and population spread. Previous research found lower rates of autism screening in rural locations in Utah, United States, which the authors proposed was due to a lack of local resources to support autistic children (5). MCHNs may therefore feel less confident talking to parents/caregivers about autism if there are fewer local services.

The overall study findings have implications for designing future autism training for nurses and other healthcare providers. For example, there may be value in customizing future training to focus on the challenges of implementing new workplace practices for older and more experienced nurses given they make up a significant proportion of the workforce, or adapting the surveillance tools used for this audience. Other opportunities include educating MCHNs about community resources for autism in their region and undertaking investigations into understanding how to better support rural and regional nurses in building their confidence to speak with parents/caregivers.

### 4.4 Strengths and limitations

This is one of the few studies of an MCHN population that examines key competencies influencing ability to identify and support families with an infant or toddler at a high likelihood for autism, and the predictors of these capabilities. Little known previous research has examined relationships with, and predictors of, confidence in speaking with parents/caregivers about autism in a healthcare professional population, with previous studies only measuring this as an outcome of training in most instances, with the exception of Shawahna and colleagues (41). Other strengths include the large sample size in the TNS, which increases confidence in generalizability of the findings.

Limitations include the small sample size in the pre/post-training comparison, which may reduce application of these findings across the broader MCHN workforce population. Future research could involve purposive sampling of a group of MCHNs for their feedback before and after training. The TNS/implementation survey matched sample cohort also had a larger proportion of nurses who worked in high SES locations in comparison to the larger TNS sample. This may have influenced results, as MCHNs might find it less daunting to discuss autism with parents/caregivers in higher SES locations who are more likely to be university educated, which is correlated with higher levels of health literacy (62).

A further limitation is the use of self-report, which can introduce bias. The use of true/false/do not know response options for the autism knowledge questionnaire also potentially introduced error through the ability to ‘guess’ the correct answer more easily. Future research could reduce this impact through use of an observer to rate competencies or the use of additional data such as the number of referrals made and whether these referrals lead to an autism diagnosis, indicating higher MCHN autism knowledge.

The length of the survey (142 questions) may have also been a disincentive for completion. As with all studies involving a convenience sample, bias was likely introduced because it is possible that only those who had a strong interest in autism completed the survey. This could be averted in future research with shorter surveys, avoiding busy times of year (where possible), and use of other methods such as interviews or observer ratings.

## 5 Conclusion

Well-child nurses such as MCHNs can play a vital role in supporting autistic children and their families through early identification and referral to appropriate diagnostic and support services. This may enable children to access early supports and services, which can improve their short-and long-term outcomes. To effectively support children and families, nurses need key competencies in areas such as autism knowledge, self-efficacy in identifying children at a high likelihood for autism, and confidence in speaking to parents/caregivers about autism. While the predictors of autism knowledge and self-efficacy, and the outcomes of autism training, have been widely researched in healthcare provider populations, there is less known research involving nurses who conduct well-baby checks, with a few exceptions (10, 25), and no known research investigating the predictors of healthcare worker confidence in speaking about autism to families. The current study has advanced knowledge in these areas by identifying predictors of these three key competencies, as well as demonstrating that targeted autism training improved competency levels in a sample of MCHNs. These results have implications for the development of future early autism training and present an opportunity for further research targeting nurses working in rural and regional areas.

## Data availability statement

The original contributions presented in the study are included in the article/supplementary material, further inquiries can be directed to the corresponding author.

## Ethics statement

The studies involving humans were approved by La Trobe University Human Research Ethics Committee, approval number: HEC18463; Department of Education and Training Ethics Committee, approval number: 2018_003864. The studies were conducted in accordance with the local legislation and institutional requirements. The participants provided their written informed consent to participate in this study.

## Author contributions

JB and MG contributed to the conception and design of the study. MG and JB collected the data. MG and KG organized the database and KG performed the statistical analyses. KG wrote the first draft of the manuscript. All authors wrote sections of the manuscript, contributed to manuscript revision, editing, read, and approved the submitted version.

## References

[1] Garg P Lillystone D Dossetor D Kefford C Chong S. An exploratory survey for understanding perceptions, knowledge and educational needs of general practitioners (GPs) regarding autistic disorders in New South Wales (NSW). Australia J Clin Diagn Res. (2014) 8:Pc01–9. doi: 10.7860/JCDR/2014/8243.4527 25177611 PMC4149117

[2] Dawson G. Early behavioral intervention, brain plasticity, and the prevention of autism spectrum disorder. Dev Psychopathol. (2008) 20:775–803. doi: 10.1017/S0954579408000370, PMID: 18606031

[3] Barbaro J Dissanayake C. Prospective identification of autism Spectrum disorders in infancy and toddlerhood using developmental surveillance: the social attention and communication study. J Dev Behav Pediatr. (2010) 31:376–85. doi: 10.1097/DBP.0b013e3181df7f3c, PMID: 20495475

[4] Warren Z McPheeters ML Sathe N Foss-Feig JH Glasser A Veenstra-VanderWeele J. A systematic review of early intensive intervention for autism Spectrum disorders. Pediatrics. (2011) 127:e1303. doi: 10.1542/peds.2011-0426, PMID: 21464190

[5] Carbone PS Norlin C Young PC. Improving early identification and ongoing Care of Children with Autism Spectrum Disorder. Pediatrics. (2016) 137:e20151850. doi: 10.1542/peds.2015-1850, PMID: 27244841

[6] Fuller EA Kaiser AP. The effects of early intervention on social communication outcomes for children with autism Spectrum disorder: a Meta-analysis. J Autism Dev Disord. (2020) 50:1683–700. doi: 10.1007/s10803-019-03927-z, PMID: 30805766 PMC7350882

[7] Corden K Brewer R Cage E. A systematic review of healthcare professionals’ knowledge, self-efficacy and attitudes towards working with autistic people. Rev J Autism Dev Disord. (2022) 9:386–99. doi: 10.1007/s40489-021-00263-w

[8] Zerbo O Massolo ML Qian Y Croen LA. A study of physician knowledge and experience with autism in adults in a large integrated healthcare system. J Autism Dev Disord. (2015) 45:4002–14. doi: 10.1007/s10803-015-2579-2, PMID: 26334872

[9] Cashin A Pracilio A Buckley T Morphet J Kersten M Trollor JN. A cross-practice context exploration of nursing preparedness and comfort to care for people with intellectual disability and autism. J Clin Nurs. (2022) 31:2971–80. doi: 10.1111/jocn.16131, PMID: 34787352

[10] Waddington H Shepherd D van der Meer L Powell-Hector N Wilson E Barbaro J. Brief report: training New Zealand well child/Tamariki Ora nurses on early autism signs using the social attention and communication surveillance-revised. J Autism Dev Disord. (2022) 52:5050–7. doi: 10.1007/s10803-021-05344-7, PMID: 34748134 PMC8574927

[11] Clarke L Fung LK. The impact of autism-related training programs on physician knowledge, self-efficacy, and practice behavior: a systematic review. Autism. (2022) 26:1626–40. doi: 10.1177/13623613221102016, PMID: 35698749

[12] Ben-Sasson A Atun-Einy O Yahav-Jonas G Lev-On S Gev T. Training physical therapists in early ASD screening. J Autism Dev Disord. (2018) 48:3926–38. doi: 10.1007/s10803-018-3668-9, PMID: 29971656

[13] Berenstein A. Predictors of screening and referral practices for autism among Canadian family physicians [doctoral dissertation]. Windsor, Ontario, Canada: University of Windsor (2012).

[14] Bordini D Lowenthal R Gadelha A Araujo Filho GM Mari Jde J Paula CS. Impact of training in autism for primary care providers: a pilot study. Braz J Psychiatry. (2015) 37:63–6. doi: 10.1590/1516-4446-2014-1367, PMID: 25372058

[15] Department of Health and Human Services (DoHaH Services) In: DoHaH Services, editor. Maternal and child health service guidelines. Melbourne, Victoria: Victoria government (2019)

[16] Department of Health and Human Services (DoHaH Services) In: DoHaH Services, editor. Maternal & Child Health Services Annual Report 2017-18. Melbourne, Victoria: Department of Health and Human Services (2019). 19.

[17] Rossiter C Fowler C Hesson A Kruske S Homer CSE Schmied V. Australian parents’ use of universal child and family health services: a consumer survey. Health Soc Care Community. (2019) 27:472–82. doi: 10.1111/hsc.12667, PMID: 30368952

[18] Public Health England In: England PH, editor. Official statistics: health visitor service delivery metrics (experimental statistics), quarter 3 2018/19 statistical commentary (April 2019). London, UK: Public Health England (2019)

[19] Takeuchi J Sakagami Y Perez RC. The mother and child health handbook in Japan as a health promotion tool: an overview of its history, contents, use, benefits, and global influence. Glob Pediatr. Health. (2016) 3:1–9. doi: 10.1177/2333794X16649884, PMID: 27336022 PMC4905145

[20] Pinto-Martin JA Souders MC Giarelli E Levy SE. The role of nurses in screening for autistic Spectrum disorder in pediatric primary care. J Pediatr Nurs. (2005) 20:163–9. doi: 10.1016/j.pedn.2005.01.004, PMID: 15933650

[21] Tebruegge M Nandini V Ritchie JJB. Does routine child health surveillance contribute to the early detection of children with pervasive developmental disorders?–an epidemiological study in Kent, UK. BMC Pediatr. (2004) 4. doi: 10.1186/1471-2431-4-4, PMID: 15053835 PMC375534

[22] Honda H Shimizu Y Nitto Y Imai M Ozawa T Iwasa M. Extraction and refinement strategy for detection of autism in 18-month-olds: a guarantee of higher sensitivity and specificity in the process of mass screening. J Child Psychol Psychiatry. (2009) 50:972–81. doi: 10.1111/j.1469-7610.2009.02055.x, PMID: 19298465

[23] Barbaro J Ridgway RN Dissanayake C. Developmental surveillance of infants and toddlers by maternal and child health nurses in an Australian community-based setting: promoting the early identification of autism Spectrum disorders. J Pediatr Nurs. (2011) 26:334–47. doi: 10.1016/j.pedn.2010.04.007, PMID: 21726784

[24] Barbaro J Sadka N Gilbert M Beattie E Li X Ridgway L. Diagnostic accuracy of the social attention and communication surveillance–revised with preschool tool for early autism detection in very Young children. JAMA Netw Open. (2022) 5:e2146415-e. doi: 10.1001/jamanetworkopen.2021.46415 35275169 PMC8917423

[25] Shrestha R Barbaro J Dissanayake C. Changes in knowledge on the signs of autism in Young children (11–30 months) among female community health volunteers in Nepal. J Autism Dev Disord. (2022) 52:219–39. doi: 10.1007/s10803-021-04944-7, PMID: 33709379

[26] Major NE Peacock G Ruben W Thomas J Weitzman CC. Autism training in pediatric residency: evaluation of a case-based curriculum. J Autism Dev Disord. (2013) 43:1171–7. doi: 10.1007/s10803-012-1662-1, PMID: 23008057

[27] Choueiri R Lindenbaum A Ravi M Robsky W Flahive J Garrison W. Improving early identification and access to diagnosis of autism Spectrum disorder in toddlers in a culturally diverse community with the rapid interactive screening test for autism in toddlers. J Autism Dev Disord. (2021) 51:3937–45. doi: 10.1007/s10803-020-04851-3, PMID: 33423215 PMC8510911

[28] Murray RE Barton EE. Training pediatricians to implement autism screening tools: a review of the literature. Rev J Autism Dev Disord. (2021) 8:108–17. doi: 10.1007/s40489-020-00206-x

[29] Allen SG Berry AD Brewster JA Chalasani RK Mack PK. Enhancing developmentally oriented primary care: an Illinois initiative to increase developmental screening in medical homes. Pediatrics. (2010) 126:S160–4. doi: 10.1542/peds.2010-1466K, PMID: 21123480

[30] Swanson AR Warren ZE Stone WL Vehorn AC Dohrmann E Humberd Q. The diagnosis of autism in community pediatric settings: does advanced training facilitate practice change? Autism. (2014) 18:555–61. doi: 10.1177/1362361313481507, PMID: 23847130

[31] Mazurek MO Curran A Burnette C Sohl K. ECHO autism STAT: accelerating early access to autism diagnosis. J Autism Dev Disord. (2019) 49:127–37. doi: 10.1007/s10803-018-3696-5, PMID: 30043354

[32] Corsano P Cinotti M Guidotti L. Paediatric nurses’ knowledge and experience of autism spectrum disorders: an Italian survey. J Child Health Care. (2020) 24:486–95. doi: 10.1177/1367493519875339, PMID: 31496265

[33] Unigwe S Buckley C Crane L Kenny L Remington A Pellicano E. GPs' confidence in caring for their patients on the autism spectrum: an online self-report study. Br J Gen Pract. (2017) 67:e445–52. doi: 10.3399/bjgp17X690449, PMID: 28483821 PMC5442960

[34] Small S. Autism Spectrum disorders (ASD): Knowledge, training, roles and responsibilities of school psychologists [doctoral dissertation]. (2012). Available at: https://digitalcommons.usf.edu/etd/4225

[35] Ries J. D. Diagnostic capability of school psychologists in the identification of autism [Doctoral dissertation]: University of Northern Colorado; (2011).

[36] Crane L Davidson I Prosser R Pellicano E. Understanding psychiatrists' knowledge, attitudes and experiences in identifying and supporting their patients on the autism spectrum: online survey. B J Psych Open. (2019) 5:e33. doi: 10.1192/bjo.2019.12, PMID: 31530309 PMC6469236

[37] Lian W Ho S Yeo C Ho L. General practitioners' knowledge on childhood developmental and behavioural disorders. Singapore Med J. (2003) 44:397–403. PMID: 14700418

[38] Bradbury K Duvall SW Armstrong K Hall TA. Survey of training experiences and clinical practice in assessment for autism spectrum disorder by neuropsychologists. Clin Neuropsychol. (2022) 36:856–73. doi: 10.1080/13854046.2021.1948610, PMID: 34308763

[39] Golnik A Ireland M Borowsky IW. Medical homes for children with autism: a physician survey. Pediatrics. (2009) 123:966–71. doi: 10.1542/peds.2008-1321, PMID: 19255027

[40] Williams ME Haranin EC. Preparation of mental health clinicians to work with children with co-occurring autism Spectrum disorders and mental health needs. J Mental Health Res Intellectual Disab. (2016) 9:83–100. doi: 10.1080/19315864.2016.1166302

[41] Shawahna R. Self-rated familiarity with autism spectrum disorders among practicing nurses: a cross-sectional study in the palestinian nursing practice. BMC Nurs. (2021) 20:241. doi: 10.1186/s12912-021-00764-3 34861861 PMC8642987

[42] Premier of Victoria. “Early Support for Children with Autism.” Published September 5, 2018. (2018). Available at: https://www.premier.vic.gov.au/early-support-children-autism

[43] Gilbert M Gore K Hawke M Barbaro J. Development, delivery, and evaluation of a training program for the early identification of autism: monitoring of social attention, interaction, and communication (MoSAIC). Front Neurol. (2023) 14:1201265. doi: 10.3389/fneur.2023.1201265 37483439 PMC10361691

[44] Productivity Commission. Child health workforce. Early childhood development workforce, research report, [internet]. Melbourne, VIC, Australia: Productivity Commission (2011).

[45] Qualtrics. Qualtrics. Utah, USA: Provo (2019).

[46] Huijg JM Gebhardt WA Dusseldorp E Verheijden MW van der Zouwe N Middelkoop BJC. Measuring determinants of implementation behavior: psychometric properties of a questionnaire based on the theoretical domains framework. Implementation Sci. (2014) 9:33. doi: 10.1186/1748-5908-9-33 PMC4000005 24641907

[47] Cane J O'Connor D Michie S. Validation of the theoretical domains framework for use in behaviour change and implementation research. Implement Sci. (2012) 7:37. doi: 10.1186/1748-5908-7-37 22530986 PMC3483008

[48] Hartley S Sikora D. Sex differences in autism Spectrum disorder: an examination of developmental functioning, autistic symptoms, and coexisting behavior problems in toddlers. J Autism Dev Disord. (2009) 39:1715–22. doi: 10.1007/s10803-009-0810-8, PMID: 19582563 PMC3590797

[49] Lawson L Joshi R Barbaro J Dissanayake C. Gender differences during toddlerhood in autism Spectrum disorder: a prospective community-based longitudinal follow-up study. J Autism Dev Disord. (2018) 48:2619–28. doi: 10.1007/s10803-018-3516-y, PMID: 29497988

[50] Postorino V Fatta L Peppo L Giovagnoli G Armando M Vicari S. Longitudinal comparison between male and female preschool children with autism spectrum disorder. J Autism Dev Disord. (2015) 45:2046–55. doi: 10.1007/s10803-015-2366-0, PMID: 25633919

[51] Faul F Erdfelder E Lang A-G Buchner A. G*power 3: a flexible statistical power analysis program for the social, behavioral, and biomedical sciences. Behav Res Methods. (2007) 39:175–91. doi: 10.3758/BF03193146, PMID: 17695343

[52] Australian Bureau of Statistics. Technical paper: Socio-economic indexes for areas (SEIFA). Canberra, Australia: Australian Bureau of Statistics (2018).

[53] Tabachnick BA Fidell LS. Cleaning up your act In: Tabachnick BA Fidell LS, editors. Using multivariate statistics. 6th ed. Uttar Pradesh, India: Pearson India Education Services (2018). 93–152.

[54] Mackintosh VH Myers BJ Goin-Kochel RP. Sources of information and support used by parents of children with autism spectrum disorders. J Develop Disab. (2005) 12:41–51.

[55] Pain H. Coping with a child with disabilities from the parents’ perspective: the function of information. Child Care Health Dev. (1999) 25:299–313. doi: 10.1046/j.1365-2214.1999.00132.x, PMID: 10399034

[56] Fragar LJ Depczynski JC. Beyond 50. Challenges at work for older nurses and allied health workers in rural Australia: a thematic analysis of focus group discussions. BMC Health Serv Res. (2011) 11:1–13. doi: 10.1186/1472-6963-11-42, PMID: 21338525 PMC3060112

[57] Ryan C Bergin M Wells J. Valuable yet vulnerable—a review of the challenges encountered by older nurses in the workplace. Int J Nurs Stud. (2017) 72:42–52. doi: 10.1016/j.ijnurstu.2017.04.006, PMID: 28456111

[58] Uthaman T Chua TL Ang SY. Older nurses: a literature review on challenges, factors in early retirement and workforce retention. Proceed Singapore Healthcare. (2016) 25:50–5. doi: 10.1177/2010105815610138, PMID: 19072190

[59] Elmensdorp S. Training physicians on the early behavioral characteristics of autism: The use of a brief, group didactic training module [doctoral dissertation]: ProQuest dissertations publishing; (2011).

[60] Jones SC Harwood V. Representations of autism in Australian print media. Disability & Society. (2009) 24:5–18. doi: 10.1080/09687590802535345

[61] Bates G. Autism in fiction and autobiography. Adv Psychiatr Treat. (2010) 16:47–52. doi: 10.1192/apt.bp.108.005660

[62] Nordahl-Hansen A Øien RA Fletcher-Watson S. Pros and cons of character portrayals of autism on TV and film. J Autism Dev Disord. (2018) 48:635–6. doi: 10.1007/s10803-017-3390-z, PMID: 29170934

[63] Durkin MS Maenner MJ Meaney FJ Levy SE DiGuiseppi C Nicholas JS. Socioeconomic inequality in the prevalence of autism spectrum disorder: evidence from a US cross-sectional study. PloS One. (2010) 5:e11551. doi: 10.1371/journal.pone.0011551, PMID: 20634960 PMC2902521

